# Standardizing definitions of the total knee alignment techniques: recommendations by the Personalized Arthroplasty Society

**DOI:** 10.1530/EOR-2024-0120

**Published:** 2025-08-04

**Authors:** The Scientific Committee from the Personalized Arthroplasty Society (PAS)

**Affiliations:** ^1^ Personalized Arthroplasty Society, Atlanta, Georgia, USA

**Keywords:** knee, arthroplasty, alignment, technique, systematic, personalized, standardization

## Abstract

- Total knee arthroplasty is a highly effective intervention for end-stage osteoarthritis, yet nearly 20% of patients report dissatisfaction with clinical outcomes.
- This dissatisfaction is often linked to intraoperative parameters, particularly whole-leg alignment and component positioning, which might play a role in ensuring both satisfaction and long-term implant survival.
- Over the past two decades, alignment techniques have progressed from systematic, two-dimensional methods focused on the frontal plane to more personalized, three-dimensional approaches.
- This evolution has introduced inconsistencies and confusion among surgeons regarding alignment techniques, terminology, and application, underscoring the need for standardized definitions that can be universally adopted.
- This work provides standardized definitions for six main knee alignment techniques to enhance communication within the scientific community, particularly in clinical research.
- While not an exhaustive analysis of each method, this effort focuses on the foundational principles of these techniques, organized using a standardized framework to facilitate comparison and improve clarity in the field.

## Introduction

Total knee arthroplasty (TKA) is a highly effective intervention for end-stage osteoarthritis, yet nearly one out of five patients report dissatisfaction with their clinical outcomes (1, 2, 3). While the causes of dissatisfaction are multifactorial, alignment has been shown to play a significant role.

Traditionally, the gold standard for coronal alignment in TKA has been to achieve a systematic postoperative neutral alignment through proximal tibial and distal femoral cuts perpendicular to the mechanical axis (4, 5). This mechanical alignment (MA) (Table 1) technique attempts to evenly distribute the load between the medial and lateral compartments, thereby reducing the incidence of implant loosening and/or wear (6). Only 0.1% of the patients scheduled for TKA naturally exhibit a proximal tibial joint line and distal femoral joint line perpendicular to the mechanical axis of the tibia and femur, respectively (7). Consequently, for most patients, MA often alters the patient’s native anatomy, necessitating ligament release(s) to compensate for alignment changes, which might further alter the intended alignment. MA gained popularity in the early days of modern TKA during the 1970s due to its simplicity, reproducibility, and compatibility with the rather trivial instrumentation at that time. In contrast, anatomical alignment (AA) (Table 2), which also aims to restore a neutral mechanical axis with an oblique joint line of 3° reflective of native knees, was more technically demanding (8).

**Table 1:** Mechanical alignment (MA).

First reported	1970’s (4)
Classification	Systematic (Fig. 1)
Guiding principle	The goal is to restore neutral alignment for all patients, regardless of their preoperative native alignment, by making proximal tibial and distal femoral cuts perpendicular to the mechanical axis. Intra-articular gaps are aimed to be equal and rectangular in both flexion and extension. This approach aims to evenly distribute the load across the implant–bone interface, promoting implant longevity and stability
Surgical workflow (example of)	The following is a generalized workflow aimed only at underlining the basic and most important steps characterizing MA. Other workflows exist (measured resection, ligament balancing, femur first, and tibia first), with multiple possible technologies providing additional information intraoperatively. The reader is provided here with the basic workflow of MA, together with relevant references that characterize the concept of MA Perform distal femoral resection perpendicular to the mechanical axis Perform proximal tibial resection perpendicular to the mechanical axis Assess extension and flexion gaps and soft-tissue tension using a spacer block or balancer Define the size of the femoral component, AP position, as well as the axial rotation relative to anatomical landmarks Complete all femoral resections Perform component trialing and final checks (e.g., stability, range of motion, patella tracking) Perform ligament releases if deemed necessary Final implantation and closure
Recommended instrumentation	Compatible with all instrumentation types (e.g. conventional mechanical instrumentation, navigation, robot, patient-specific instrumentation (PSI), and ligament tensioner/balancer)
Perceived advantages and limitations	Advantages:
	Even load distribution between the medial and lateral compartments: this minimizes wear and reduces the risk of potential component loosening
	Gold standard for decades: numerous studies have reported satisfactory clinical outcomes and long-term survival of implants using this approach
	Limitations:
	Native varus in one-third of the population: MA could impact the medial side, requiring soft-tissue release (medial collateral ligament). ‘Forcing neutral alignment in patients with native varus or valgus limb alignment requires significant soft-tissue release’
	Perpendicular positioning of the tibial and femoral components to the mechanical axis can lead to a situation different from the native knee, with abnormal joint line obliquity and alteration of normal knee biomechanics, which could negatively influence clinical results in TKA
Clinical results	‘Excellent survivorship’ for TKA patients at a mean 20-year follow-up (19- to 25-year follow-up), although the functional outcomes were overall disappointing (22)
	‘The study did not demonstrate a statistically significant or clinically meaningful difference in the 20-year survival between knees that were mechanically aligned and those that were outliers’ (23)
Key supportive references	(4, 5, 24, 25, 26, 27)

**Figure 1:**
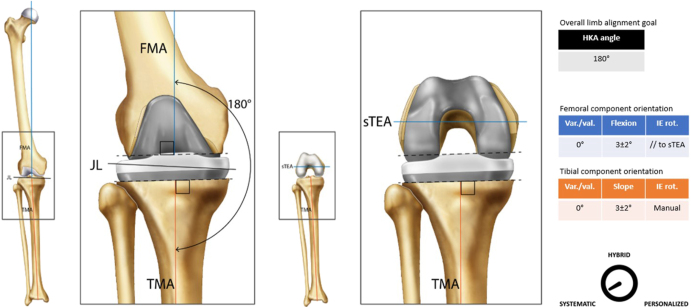
Usual femoral and tibial cut parameters associated with MA. The femoral (in blue) and tibial (in orange) cut parameters, the suggested references, and the overall limb alignment goal should be perceived as typical recommendations for each considered alignment technique. Therefore, they are subject to fluctuate depending on the user’s preferences. The joint line (JL) represents the orientation of the coronal femorotibial joint line in extension on a long leg standing X-ray. The gauge should be understood as a graphical illustration intended to express the personalization of a given alignment technique from a directional perspective.

**Figure 2:**
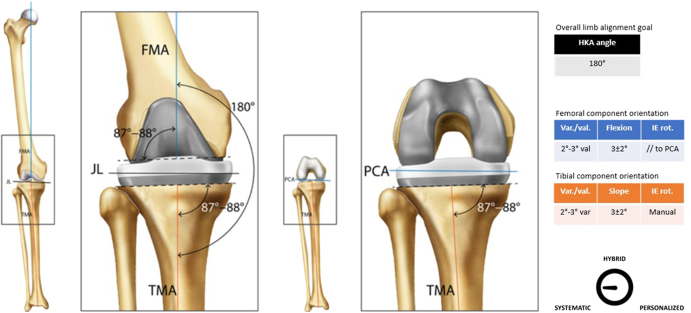
Usual femoral and tibial cut parameters associated with AA.

**Table 2:** Anatomical alignment (AA).

First reported	1980’s (28)
Classification	Systematic (Fig. 2)
Guiding principle	The goal is to restore neutral alignment for all patients, irrespective of preoperative alignment, while preserving the natural obliquity of the joint line, typically 3°, consistent with the mean population value of native knees
Surgical workflow (example of)	Perform distal femoral resection with a 2°–3° valgus relative to the mechanical axis Perform proximal tibial resection with a 2°–3° varus relative to the mechanical axis Use a spacer block or balancer to assess extension/flexion gap and soft-tissue tension Define the size of the femoral component, AP position, as well as the axial rotation relative to anatomical landmarks (e.g. PCA for the axial rotation) Complete all femoral resections Perform component trialing and final checks (e.g. stability, range of motion, patella tracking) Perform ligament releases if deemed necessary Final implantation and closure
Recommended instrumentation	Compatible with all instrumentation types (e.g. conventional mechanical instrumentation, navigation, robot, patient-specific instrumentation (PSI))
Perceived advantages and limitations	Advantages:
	Increased implant stability throughout the full range of motion: less ligament release is required during the procedure with AA compared to MA
	Preliminary studies have reported good clinical outcomes, but with short-term follow-ups
	Limitations:
	Wide individual variation in limb alignment: the neutral condition can be ‘unnatural’ for many patients Inadvertent over-resection risk: over-resecting more than 3° in the proximal tibial cut may lead to excessive varus of the tibial implant, which is associated with premature component failure in TKA. Technical difficulties: performing the varus cut on the tibia precisely and reproducibly is challenging using conventional instrumentation Lack of reproducibility and long-term results: due to these issues, this alignment technique was progressively abandoned
Clinical results	56 TKA with MA vs 61 TKA with AA: at the 24-months follow-up, no significant differences were observed in ROM, HSS, and WOMAC scores (*P* > 0.05). The study concluded that both alignment techniques provide comparable clinical outcomes after primary TKA (29)
Key supportive references	(28, 30)

To better preserve a patient’s native anatomy, alternative techniques with different alignment targets have been developed. The adjusted mechanical alignment (aMA) (Table 3) technique incorporates the concept of constitutional alignment, recognizing that a substantial part of the population naturally deviates from neutral alignment. aMA fine-tunes the frontal orientation of the tibia and/or the femoral component to preserve aspects of the patient’s frontal native deformity (varus or valgus) (9, 10, 11). This approach aims to achieve proper ligament balancing through the bone cuts rather than subsequent ligament releases.

**Table 3:** Adjusted mechanical alignment (aMA).

First reported	2012 (9, 10)
Classification	Hybrid (Fig. 3)
Guiding principle	The goal is to maintain aspects of the frontal native deformity (generally up to 3°) by fine-tuning the frontal orientation of the tibia and/or the femoral component to achieve proper ligament balancing through a tibial and/or femoral resection/correction instead of the typical soft-tissue releases
Surgical workflow (example of)	Define the required adjustment of the proximal tibial cut and/or distal femoral cut in the frontal plane to preserve mild native deformity within a limit of ±3° Perform the distal femoral cut based on the defined frontal alignment Perform the 4-in-1 cuts based on bony landmarks for the setup of the axial rotation Perform proximal tibial resection based on the defined frontal alignment Using a spacer block or balancer to assess extension/flexion gap and soft-tissue tension Perform component trialing and final checks (e.g. stability, range of motion, and patella tracking) Perform ligament releases if deemed necessary Final implantation and closure
Recommended instrumentation	Compatible with all instrument families: conventional mechanical instruments, navigation, robot, and PSI. The use of navigation or robot is recommended to improve the precision of execution of the planned cuts and avoid outliers. When used with conventional mechanical instrumentation, preoperative planning based on long-leg X-ray is recommended to assess the deformity in the frontal plane. Similarly, a balancer/ligament tensioner is recommended
Perceived advantages and limitations	Advantages:
	Reduces soft-tissue releases: compared to traditional MA, the need for soft-tissue release is minimized
	Streamlined proximal tibial cut: follows the MA method, simplifying the procedure
	Compatibility with various surgical workflows: works with different bone preparation sequences and types of instrumentation
	Better clinical and functional outcomes: shows improved scores compared to MA at short follow-up
	Limitations:
	Risk of HKA outlier: when using conventional mechanical instrumentation, there is a risk of HKA outliers
	Comparative superiority: the superiority of the aMA in clinical outcomes is only established when compared to MA
	Lack of long-term clinical evidence
	No focus on the flexion gap
Clinical results	143 PS TKA with aMA: patients with preoperative varus had better clinical and functional outcome scores if the alignment was left in mild varus, as compared with patients with an alignment correction to neutral (11)
	75 TKA with aMA and MA: at the 24-months follow-up, it was found that the aMA patients were associated with significantly better clinical outcomes when assessed using the Knee Society Score, the Forgotten Joint Score, and the High Flexion Knee Score (31)
	40 TKA with aMA vs 40 TKA with restricted inverse KA (iKA): both restricted iKA and aMA grant comparable clinical outcomes at 12-month follow-up, though a greater proportion of knees operated on by restricted iKA achieved the PASS thresholds for OKS and satisfaction (15)
Key supportive references	(9, 10, 11, 31, 32, 33)

**Figure 3:**
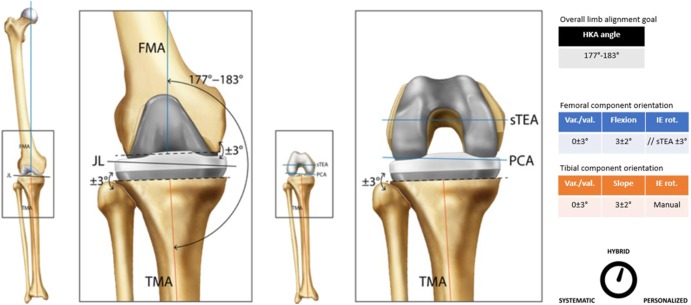
Usual femoral and tibial cut parameters associated with aMA.

**Figure 4:**
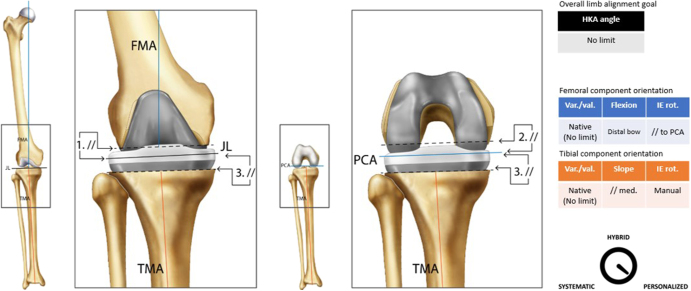
Usual femoral and tibial cut parameters associated with KA. Numbers 1, 2, and 3 express the order of the bone cuts: 1. distal femoral cut, 2. final femoral cuts, and 3. proximal tibial cut. Symbol//shows that, after taking cartilage (and bone loss) into account, equal cuts are performed on both distal and posterior femoral condyles, as well as equal medial and lateral proximal tibial cuts.

Kinematic alignment (KA) (Table 4) represents a more personalized approach, striving to restore the knee’s native anatomy by accounting for cartilage and bone loss during bone-cut planning (12). However, in cases of severe lower limb deformity, KA may result in implant alignment traditionally considered unsafe. To address this concern, restricted kinematic alignment (rKA) (Table 5) reproduces the patient’s native knee anatomy while ensuring alignment remains within a proposed safe range, avoiding extreme or pathological anatomies (7). Similarly, inverse kinematic alignment (iKA) prioritizes restoring native tibial anatomy first (measured resection) and achieves gap balancing through adjustments of the femoral implant position and/or orientation (13).

**Table 4:** Unrestricted kinematic alignment (KA).

First reported	2008 (12)
Classification	Personalized (Fig. 4)
Guiding principle	The goal is to recreate knee axis while performing a resurfacing operation of the knee by placing the implant surface at the same level as the native joint to establish near-normal kinematics
Surgical workflow (example of)	Placement of the implants into the position of the native joint is accomplished using unrestricted guides The femoral cuts are completed and measured with a caliper. The thickness of a kerf (saw thickness) and wear are added to the measurements The tibia is resected similarly and aligned to the native tibia varus angle and the native medial posterior slope. In the case of tibial wear, depth is calculated to achieve a resection that places the implant surface at the same height as the native cartilaginous surface. The posterior slope is set to replicate the native slope if a CR implant is used Except in the case of severe flexion contracture, where the posterior capsule may be released, no collateral ligament releases are required. Proper balance is achieved through a replication of the native knee ligamentous tension by this ‘resurfacing’ procedure. Patella tracking is theoretically normal, unrestricted KA restores the patient’s native femoral angulation and q angle Balance is then assessed with trials placed. In full extension, the goal is negligible varus/valgus laxity and minimal rotation. The medial compartment should be stable throughout the arc of flexion with 1–2 mm of laxity (up to 3 mm in case of hyperlaxity). The lateral compartment tends to be laxer, and there exists a high inter-individual range Failing to restore the alignment, the gaps, and/or the rotation (usually in case of tibial bone loss) may require a recut of the tibia. Except if the bone loss precludes the proper assessment of the native anatomy, bone loss should not be considered a contraindication for unrestricted KA
Recommended instrumentation	Compatible with all instrument families. Caliper checks required
Perceived advantages and limitations	Advantages:
	Reconstructs specific patients’ native alignment: mimics the ligamentous tensions and rotational moments as close to natural
	Streamlined femur, reproducible, exactly duplicates native coronal varus/valgus of distal femur
	Restoring precise native DLFA and posterior condylar line
	Intraoperative checks measure the thickness of the cuts, guiding through surgery
	Limitations:
	Actual implants have been designed based on MA, and their use for KA might alter the patellofemoral joint kinematics in some patients
	In a specific knee phenotype (CPAK III), placing a standard femoral implant in extensive valgus can alter patella tracking relative to the quadriceps line of force (34)
	KA generates a substantial amount of trochlear orientation outliers when lateral trochlear inclination, sulcus angle, and anterior trochlear line angle are considered (35, 36). Surgeons should be aware of all relevant implant-related parameters and judge their alignment strategy based on patient-specific anatomy (37)
Clinical results	41 CR TKA with KA performed with PSI vs 41 CR TKA treated with MA using measured resection and manual instrumentation: there were no inclusion or postoperative correction restrictions. A 2-year follow-up of the same cohort, published in 2014, reported 8° more flexion in the KA cohort, and a 3.2 and 4.9 odds ratio of having a pain-free knee based on the Oxford and WOMAC pain subsections, respectively, compared with the MA group (38)
	100 CR TKA with KA performed with PSI vs 100 CR TKA treated with MA using measured resection and manual instrumentation: follow-up was at 1 year. There were inclusion restrictions, as patients were excluded when the preoperative varus and valgus deformities were greater than ±10°. Ligaments were not released in the KA cohort. The KA cohort had significantly higher KSS and WOMAC scores (39)
	Simultaneous bilateral CR TKA in 41 patients with KA and MA in opposite knees using navigation: valgus knee deformities were excluded. There was a correction restriction, as the postoperative HKA angle limits of the KA cohort were limited from 6° varus to 3° valgus. Follow-up was at 2 years. Significantly more participants preferred their KA joint, although clinical outcomes were equivalent. Fewer releases were required using the KA technique. Participants were visually insensitive to modest HKA asymmetry (40)
Key supportive references	(12, 38, 39, 41, 42, 43, 44)

**Table 5:** Restricted kinematic alignment (rKA).

First reported	2017 (7)
Classification	Personalized (Fig. 5)
Guiding principle	Restricted kinematic alignment combines the principles of kinematic alignment with predefined safety boundaries to prevent extreme or pathological alignment patterns while preserving the patient’s native knee anatomy
Surgical workflow (example of)	An rKA algorithm following the leading principles has been proposed:
	Patients with both mMPTA and mLDFA ≤5° and an HKA ≤3°, no bone cut adjustment is needed, and an unrestricted KA technique is performed
	When the patient’s anatomy falls outside the rKA limits, as rKA believes that the femoral flexion axis plays a more significant role in knee kinematics, then the priority is to preserve the femoral anatomy and to perform most modifications from the tibia
	If the mMPTA and mLDFA are <5° but the HKA is >3° varus (in 8% of cases) or>3° valgus (in 7% of cases), then the mMPTA should be corrected to fall within 3° of HKA If the mMPTA and/or mLDFA >5°, in varus knees, the mMPTA needs to be adjusted to 5°, while in valgus knees, the mLDFA should be brought to 5°
	⁃ If those resections bring the HKA ≤3°, the rKA objective is achieved
	⁃ If the resultant aHKA is >3°, the previously unchanged parameter should be corrected, namely, mLDFA in varus knees and mMPTA in valgus, until the HKA is ≤3°
	Ligamentous releases are rarely needed in cases with anatomic modifications of <3°. In more significant corrections, minimal releases can be done, keeping the goal of native ligament balance reproduction (not aiming for isometry)
	To preserve femoral anatomy, rKA aims to resurface the posterior condyles. Using a posterior referencing set to neutral rotation, the implant thickness on both posterior condyles will be resected without modifying femoral rotation. The tibial component’s rotation is set by its alignment with the femoral trial component, keeping the knee in 10° of flexion
Recommended instrumentation	Since many patients will require anatomic modification to fit within rKA boundaries, rKA is ideally performed with patient-specific instrumentation (PSI), intraoperative computer navigation, or robotic assistance. If the resected pieces do not match the computer plan, or ligament laxities assessed with trial implants fall outside the expected native ligament laxity range, the resection accuracy can be confirmed by caliper measurement, and cut adjustment is performed when needed
Perceived advantages and limitations	Advantages:
	Reproduce patient’s native knee anatomy within a safe range
	Avoids reproducing pathological anatomies and may prevent related implant failures
	Limitations:
	Almost 50% of the TKA patients require some anatomical modifications
	10% of cases with outlier anatomies will require soft-tissue releases to obtain ligament stability
	Precision tools (PSI, NAV, or robot) are essential to perform the technique
Clinical results	100 CR TKA with rKA: no revision for aseptic loosening at 49 (32–60) months of follow-up. It also demonstrated excellent osseointegration of the implants. The WOMAC, KOOS, and Forgotten Joint scores were similar to those reported for cemented KA TKAs (45)
	100 CR TKA with rKA compared to MA: gait patterns of patients operated on with rKA were significantly closer to healthy controls than in MA TKAs. In addition, the rKA group presented a significantly higher postoperative KOOS score when compared to the MA group (74 vs 61, *P* = 0.034) (46)
	121 TKA with rKA vs 115 matched TKA with MA: 93% of rKA patients were either very satisfied or satisfied compared to 81% in MA TKAs (*P* < 0.006). At mean follow-up of 17 months, the mean PROMs were significantly better in the rKA group than in the neutral MA group (47)
Key supportive references	(7, 45, 46, 47, 48, 49, 50, 51)

**Figure 5:**
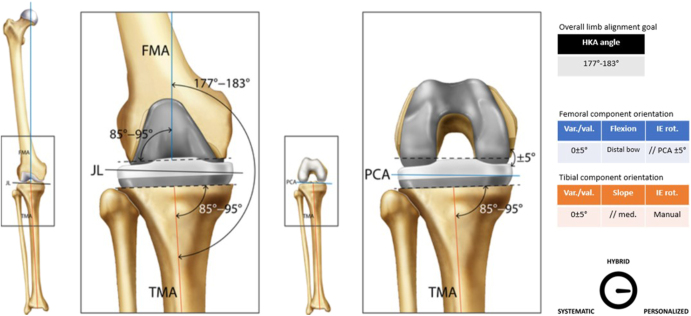
Usual femoral and tibial cut parameters associated with rKA.

**Figure 6:**
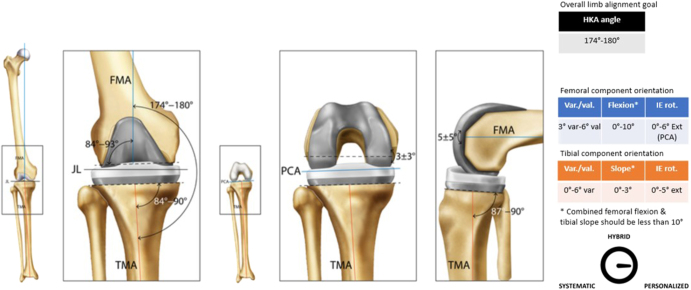
Usual femoral and tibial cut parameters associated with FA.

Advancements in surgical technologies, such as computer navigation and robotic-assisted systems, along with ligament assessment, enable personalized implant positioning and alignment plans tailored to the patient’s native anatomy, size, and soft-tissue characteristics. The functional alignment (FA) (Table 6) technique integrates measured resections with three-dimensional gap balancing, aiming to reproduce native knee anatomy within predefined boundaries (14, 15, 16, 17). Because of the large number of parameters assessed, FA is preferably performed using robotic and computer-assisted navigation technologies. FA can adapt to various starting techniques, such as planning cuts perpendicular to the mechanical axis or incorporating native tibial joint line obliquity. It also allows for personalized adjustments, such as measuring the ligamentous tension before any bone cuts or after the completion of the proximal tibial cut, allowing for a more complete removal of the osteophytes.

**Table 6:** Functional alignment (FA) (aka. Functional positioning).

First reported	2020 (14)
Classification	Personalized (Fig. 6)
Guiding principle	The goal is to reconstruct 3-dimensional native alignment while achieving balanced flexion–extension gaps and soft-tissue tension, currently within given boundaries (16, 17)
Surgical workflow (example of pre-resection workflow)	Preplan TKA implant position using CT scan or intraoperatively using bone morphing Remove medial and lateral osteophytes Acquire arthritic knee alignment in full extension. Then, correct the knee alignment to its likely native position. The soft-tissue envelope of the arthritic knee is collected by stressing the collateral ligaments in extension and in flexion (90°) The robotic or computer navigation system produces a balance evaluation, which enables the surgeon to optimally balance the TKA Small positional changes are made on the TKA planning screen from the preplanning to optimally balance the TKA Optimal balance is defined as having a balanced extension gap (<2 mm mediolateral laxity). The medial side of the knee is balanced through a full ROM, and the 2 mm more lateral laxity is applied in flexion to enable the TKA to roll back laterally in flexion. Because functional alignment aims to maintain the overall joint line obliquity, extension laxity tends to be minimal (≤2 mm). Furthermore, flexion laxity tends to be much more variable between individuals, and with surgeons having preferred targets for lateral laxity. Currently, the optimal accepted laxity in flexion, and whether we should also accept slight (<2 mm) laxity in extension, remains unclear and still represents a topic of intense discussion. For this reason, surgeons should consult the up-to-date literature, company recommendations, and consider the type of insert used in order to decide how much laxity is safe for their patients Trochlear groove restoration is controlled, notably when image-based technology is used. If a compromise must be made, the trochlea is favored over flexion balance All cuts are made with either a robotic or computer navigation system Balance is then assessed with trials placed
Perceived advantages and limitations	Advantages:
	Restoration of the patient’s specific native alignment with small modifications to enable a well-balanced TKA
	Restoration of the soft tissue envelope so the natural ligamentous tensions are maintained
	Restoration of the trochlear groove
	Limitations:
	Optimal implant positioning is determined before any posterior osteophytes, tight posterior capsule, and menisci are removed (pre-resection workflow). Later removal of these may affect the balance of the TKA
	This technique requires either a robotic or a computer navigation system to intraoperatively morph the bony anatomy and soft-tissue envelope of the knee to provide intraoperative data to create a balance curve to evaluate the balance consequences of the TKA implant positioning
Clinical results	30 TKA with robotic FA: FA achieves balanced mediolateral soft-tissue tension through the arc of knee flexion, as assessed using intraoperative pressure-sensor technology (52)
	122 TKA: a kinematically placed femoral component led to positioning considered unsafe in over 13% of cases. A functionally placed femoral component most closely restored trochlea depth in all three positions of flexion (16)
	60 TKA with robotic FA versus 60 TKA with conventional MA at 2 years’ FU: greater functional outcomes and satisfaction in the robotic FA group (53)
	300 CR TKA with FA, MA, and KA: FA more consistently achieves a balanced total knee arthroplasty than either MA or KA before undertaking soft-tissue release (54)
	100 PS TKA with robotic FA versus 100 PS TKA with conventional rKA at 1-year FU: significantly higher Forgotten Joint Score (FJS) in the robotic FA group (55)
Key supportive references	(14, 16, 17, 52, 53, 54, 55)

The debate surrounding alignment strategies for TKA is more active than ever within the orthopedic community. The proliferation of alignment philosophies and their numerous variants has introduced an array of technical acronyms, leading to a dynamic yet inconsistent adoption among surgeons. Modern personalized alignment techniques are inherently more complex than historical approaches, requiring surgeons to navigate preoperative and intraoperative decision trees that incorporate multiple inputs throughout the surgical workflow. Moreover, alignment techniques have evolved from two-dimensional (2D) references limited to the frontal plane to three-dimensional (3D) frameworks that evaluate femoral and tibial resections across the frontal, sagittal, and axial planes. In addition to considering leg alignment guidance, modern alignment techniques consider the soft-tissue laxity too, which tends to be a subjective rather than objective parameter. This complexity has resulted in inconsistency in terminology, application, and understanding of the various techniques.

Although several recent studies provide overviews of the most common techniques (18, 19, 20), consensus about a concise definition for each technique has yet to be reached (21). There is a clear need for universally accepted definitions that the surgical community can reliably adopt to foster clarity, improve collaboration, and enable meaningful comparisons of outcomes.

The objective of the Personalized Arthroplasty Society (PAS) was to reach a consensus on standardized definitions for TKA alignment techniques by partnering with its active members. In developing these definitions, the PAS task force adhered to the following guiding principles:

-Unbiased: no endorsements of any particular technique,
-Pragmatic and straightforward approaches: focused on the ease of understanding,
-Accurate: partnered with key initiators/experts of the alignment techniques,
-Global: developed and approved by the PAS community during an international survey initiative on the most conflictual elements of TKA alignment principles.

The included tables and figures provide detailed summaries and visual representations of all six alignment techniques. The tables offer an organized overview of key attributes for each technique, including their historical development, classification, guiding principles, surgical workflow examples, decision-making processes, recommended instrumentation, perceived advantages and limitations, clinical outcomes, and key supporting references. These tables serve as a quick reference for comparing techniques, highlighting their unique features and practical applications. Complementing the tables, the figures illustrate the alignment concepts and surgical workflows, offering visual clarity to enhance understanding. Together, these tables and figures provide a cohesive framework to support the clinical and research community in adopting standardized definitions and evaluating alignment strategies in TKA.

## Discussion/conclusion

The primary aim of this work was to provide clear, standardized definitions for six main knee alignment techniques to enhance communication within the scientific and clinical research community. Rather than offering an exhaustive analysis of each knee alignment technique, the focus was on presenting the fundamental principles agreed upon by the PAS Scientific Committee in a concise and accessible format. While numerous additional knee alignment techniques exist, this work serves as a foundational reference for understanding and comparing these techniques.

The increasing adoption of enabling technologies is expected to further support the standardization of alignment techniques. These advanced tools offer several key benefits:

- Consistent surgical workflow: enabling technologies follow a predefined workflow tailored by the surgeon, ensuring consistency across surgical steps.
- Soft-tissue characterization: they provide the ability to characterize the soft-tissue envelope at discrete flexion angles or throughout the range of motion, incorporating this information into precise surgical planning.
- Accurate execution: these tools enable more reliable execution by consistently delivering fewer alignment outliers compared to conventional implantation methods. Nonetheless, advanced technologies still depend on the precise acquisition of anatomical landmarks. This step requires careful consideration, as even standardized landmark selection can lead to the implantation variability commonly observed with conventional techniques.
- Comprehensive recording: by documenting every surgical step, they provide insights into intraoperative intricacies and create a continuum of care from preoperative to postoperative follow-ups.

By standardizing knee alignment techniques and leveraging intraoperative data, these advancements lay the groundwork for identifying best practices in personalized alignment for TKA patients.

In order to facilitate rapid dissemination and adoption of technological advancements within the global orthopedic community, we propose to adopt this set of definitions on the most prevalent alignment techniques, their key alignment parameters, and alignment goals. By unifying our surgical language, we will i) accelerate knowledge flow from high- to low-income countries, ii) aid surgeons in understanding the current literature, and iii) prepare the groundwork for a future unified conceptual framework on knee joint alignment.

## ICMJE Statement of Interest

There is no conflict of interest that could be perceived as prejudicing the impartiality of the research reported.

## Funding Statement

This work did not receive any specific grant from any funding agency in the public, commercial, or not-for-profit sector.

## References

[1] Bourne RB, Chesworth BM, Davis AM, et al. Patient satisfaction after total knee arthroplasty: who is satisfied and who is not? Clin Orthop Relat Res 2010 468 57–63. (10.1007/s11999-009-1119-9) 19844772 PMC2795819

[2] Dunbar MJ, Richardson G & Robertsson O. I can’t get no satisfaction after my total knee replacement: rhymes and reasons. Bone Joint Lett J 2013 95-B (11 Supplement A) 148–152. (10.1302/0301-620x.95b11.32767) 24187375

[3] Bryan S, Goldsmith LJ, Davis JC, et al. Revisiting patient satisfaction following total knee arthroplasty: a longitudinal observational study. BMC Musculoskelet Disord 2018 19 423. (10.1186/s12891-018-2340-z) 30497445 PMC6267049

[4] Freeman MA, Swanson SA & Todd RC. Total replacement of the knee using the Freeman-Swanson knee prosthesis. Clin Orthop Relat Res 1973 94 153–170. (10.1097/00003086-197307000-00020) 4743445

[5] Insall J, Scott WN & Ranawat CS. The total condylar knee prosthesis. A report of two hundred and twenty cases. J Bone Joint Surg Am 1979 61 173–180. (10.2106/00004623-197961020-00003) 422602

[6] Ritter MA, Davis KE, Meding JB, et al. The effect of alignment and BMI on failure of total knee replacement. J Bone Joint Surg Am 2011 93 1588–1596. (10.2106/jbjs.j.00772) 21915573

[7] Almaawi AM, Hutt JRB, Masse V, et al. The impact of mechanical and restricted kinematic alignment on knee anatomy in total knee arthroplasty. J Arthroplast 2017 32 2133–2140. (10.1016/j.arth.2017.02.028) 28302462

[8] Hungerford DS, Kenna RV & Krackow KA. The porous-coated anatomic total knee. Orthop Clin North Am 1982 13 103–122. (10.1016/s0030-5898(20)30270-4) 7063184

[9] Bellemans J. Neutral mechanical alignment: a requirement for successful TKA: opposes. Orthopedics 2011 34 e507–e509. (10.3928/01477447-20110714-41) 21902146

[10] Bellemans J, Colyn W, Vandenneucker H, et al. The Chitranjan Ranawat award: is neutral mechanical alignment normal for all patients? The concept of constitutional varus. Clin Orthop Relat Res 2012 470 45–53. (10.1007/s11999-011-1936-5) 21656315 PMC3237976

[11] Vanlommel L, Vanlommel J, Claes S, et al. Slight undercorrection following total knee arthroplasty results in superior clinical outcomes in varus knees. Knee Surg Sports Traumatol Arthrosc 2013 21 2325–2330. (10.1007/s00167-013-2481-4) 23552665

[12] Howell SM, Kuznik K, Hull ML, et al. Results of an initial experience with custom-fit positioning total knee arthroplasty in a series of 48 patients. Orthopedics 2008 31 857–863. (10.3928/01477447-20080901-15) 18814593

[13] Winnock de Grave P, Kellens J, Luyckx T, et al. Inverse kinematic alignment for total knee arthroplasty. Orthop Traumatol Surg Res 2022 108 103305. (10.1016/j.otsr.2022.103305) 35513224

[14] Oussedik S, Abdel MP, Victor J, et al. Alignment in total knee arthroplasty. Bone Joint Lett J 2020 102-B 276–279. (10.1302/0301-620x.102b3.bjj-2019-1729) 32114811

[15] Winnock de Grave P, Luyckx T, Claeys K, et al. Higher satisfaction after total knee arthroplasty using restricted inverse kinematic alignment compared to adjusted mechanical alignment. Knee Surg Sports Traumatol Arthrosc 2022 30 488–499. (10.1007/s00167-020-06165-4) 32737528 PMC8866329

[16] Shatrov J, Battelier C, Sappey-Marinier E, et al. Functional alignment philosophy in total knee arthroplasty – rationale and technique for the varus morphotype using a CT based robotic platform and individualized planning. SICOT J 2022 8 11. (10.1051/sicotj/2022010) 35363136 PMC8973302

[17] Shatrov J, Foissey C, Kafelov M, et al. Functional alignment philosophy in total knee arthroplasty-rationale and technique for the valgus morphotype using an image based robotic platform and individualized planning. J Pers Med 2023 13 212. (10.3390/jpm13020212) 36836446 PMC9961945

[18] Lustig S, Sappey-Marinier E, Fary C, et al. Personalized alignment in total knee arthroplasty: current concepts. SICOT J 2021 7 9. (10.1051/sicotj/2021021) 33812467 PMC8019550

[19] Begum FA, Kayani B, Magan AA, et al. Current concepts in total knee arthroplasty. Bone Jt Open 2021 2 397–404. (10.1302/2633-1462.26.bjo-2020-0162.r1) 34139884 PMC8244789

[20] Karasavvidis T, Pagan Moldenhauer CA, Haddad FS, et al. Current concepts in alignment in total knee arthroplasty. J Arthroplast 2023 38 (7 Supplement 2) S29–S37. (10.1016/j.arth.2023.01.060) 36773657

[21] Walker LC, Clement ND, Ghosh KM, et al. What is a balanced knee replacement? EFORT Open Rev 2018 3 614–619. (10.1302/2058-5241.3.180008) 30697441 PMC6335603

[22] Patil S, McCauley JC, Pulido P, et al. How do knee implants perform past the second decade? Nineteen- to 25-year followup of the press-fit condylar design TKA. Clin Orthop Relat Res 2015 473 135–140. (10.1007/s11999-014-3792-6) 25082622 PMC4390935

[23] Abdel MP, Oussedik S, Parratte S, et al. Coronal alignment in total knee replacement: historical review, contemporary analysis, and future direction. Bone Joint Lett J 2014 96-B 857–862. (10.1302/0301-620x.96b7.33946) 24986936

[24] Ritter MA. The anatomical graduated component total knee replacement: a long-term evaluation with 20-year survival analysis. J Bone Joint Surg Br 2009 91 745–749. (10.1302/0301-620x.91b6.21854) 19483226

[25] Roussot MA, Vles GF & Oussedik S. Clinical outcomes of kinematic alignment versus mechanical alignment in total knee arthroplasty: a systematic review. EFORT Open Rev 2020 5 486–497. (10.1302/2058-5241.5.190093) 32953134 PMC7484715

[26] Diduch DR, Insall JN, Scott WN, et al. Total knee replacement in young, active patients. Long-term follow-up and functional outcome. J Bone Joint Surg Am 1997 79 575–582. (10.2106/00004623-199704000-00015) 9111404

[27] Rodriguez JA, Bhende H & Ranawat CS. Total condylar knee replacement: a 20-year followup study. Clin Orthop Relat Res 2001 388 10–17. (10.1097/00003086-200107000-00004) 11451106

[28] Hungerford DS & Krackow KA. Total joint arthroplasty of the knee. Clin Orthop Relat Res 1985 192 23–33. (10.1097/00003086-198501000-00004) 3967427

[29] Yim JH, Song EK, Khan MS, et al. A comparison of classical and anatomical total knee alignment methods in robotic total knee arthroplasty: classical and anatomical knee alignment methods in TKA. J Arthroplast 2013 28 932–937. (10.1016/j.arth.2013.01.013) 23540531

[30] Cherian JJ, Kapadia BH, Banerjee S, et al. Mechanical, anatomical, and kinematic axis in TKA: concepts and practical applications. Curr Rev Musculoskelet Med 2014 7 89–95. (10.1007/s12178-014-9218-y) 24671469 PMC4092202

[31] Hommel H, Tsamassiotis S, Falk R, et al. [Adjusted mechanical alignment: operative technique-tips and tricks]. Orthopä 2020 49 562–569. (10.1007/s00132-020-03929-1) 32494903

[32] Zheng K, Sun H, Zhang W, et al. Mid-term outcomes of navigation-assisted primary total knee arthroplasty using adjusted mechanical alignment. Orthop Surg 2023 15 230–238. (10.1111/os.13595) 36440506 PMC9837234

[33] Pan Y, Jiang B, Li Y, et al. Alignment analysis of brainlab knee 3 navigation-guided total knee arthroplasty using the adjusted mechanical method. Front Surg 2022 9 1040025. (10.3389/fsurg.2022.1040025) 36425888 PMC9679003

[34] Howell SM, Sappey-Marinier E, Niesen AE, et al. Better forgotten joint scores when the angle of the prosthetic trochlea is lateral to the quadriceps vector in kinematically aligned total knee arthroplasty. Knee Surg Sports Traumatol Arthrosc 2023 31 5438–5445. (10.1007/s00167-023-07598-3) 37792084

[35] Klasan A, Anelli-Monti V, Putnis SE, et al. The effect of different alignment strategies on trochlear orientation after total knee arthroplasty. Knee Surg Sports Traumatol Arthrosc 2024 32 1734–1742. (10.1002/ksa.12178) 38606595

[36] Hess S, Chelli S, Leclercq V, et al. Three-compartment phenotype concept of total knee arthroplasty alignment: mismatch between distal femoral, posterior femoral, and tibial joint lines. J Arthroplast 2025 40 2023–2034. (10.1016/j.arth.2025.02.015) 40049560

[37] Hirschmann MT, Khan ZA, Sava MP, et al. Definition of normal, neutral, deviant and aberrant coronal knee alignment for total knee arthroplasty. Knee Surg Sports Traumatol Arthrosc 2024 32 473–489. (10.1002/ksa.12066) 38293728

[38] Dossett HG, Estrada NA, Swartz GJ, et al. A randomised controlled trial of kinematically and mechanically aligned total knee replacements: two-year clinical results. Bone Joint Lett J 2014 96-B 907–913. (10.1302/0301-620x.96b7.32812) 24986944

[39] Calliess T, Bauer K, Stukenborg-Colsman C, et al. PSI kinematic versus non-PSI mechanical alignment in total knee arthroplasty: a prospective, randomized study. Knee Surg Sports Traumatol Arthrosc 2017 25 1743–1748. (10.1007/s00167-016-4136-8) 27120192

[40] McEwen PJ, Dlaska CE, Jovanovic IA, et al. Computer-assisted kinematic and mechanical axis total knee arthroplasty: a prospective randomized controlled trial of bilateral simultaneous surgery. J Arthroplast 2020 35 443–450. (10.1016/j.arth.2019.08.064) 31591010

[41] Howell SM, Shelton TJ & Hull ML. Implant survival and function ten years after kinematically aligned total knee arthroplasty. J Arthroplast 2018 33 3678–3684. (10.1016/j.arth.2018.07.020) 30122435

[42] Howell SM. Calipered kinematically aligned total knee arthroplasty: an accurate technique that improves patient outcomes and implant survival. Orthopedics 2019 42 126–135. (10.3928/01477447-20190424-02) 31099877

[43] Rivière C, Iranpour F, Harris S, et al. The kinematic alignment technique for TKA reliably aligns the femoral component with the cylindrical axis. J Orthop Traumatol: Surg Res 2017 103 1069–1073. (10.1016/j.otsr.2017.06.016) 28870873

[44] Rivière C, Iranpour F, Harris S, et al. Differences in trochlear parameters between native and prosthetic kinematically or mechanically aligned knees. Orthop Traumatol Surg Res 2018 104 165–170. (10.1016/j.otsr.2017.10.009) 29223778

[45] Laforest G, Kostretzis L, Kiss MO, et al. Restricted kinematic alignment leads to uncompromised osseointegration of cementless total knee arthroplasty. Knee Surg Sports Traumatol Arthrosc 2022 30 705–712. (10.1007/s00167-020-06427-1) 33452903 PMC8866348

[46] Blakeney WG & Vendittoli PA. Restricted kinematic alignment: the ideal compromise?. In Personalized Hip and Knee Joint Replacement [Internet], pp 197–206. Eds C Rivière, PA Vendittoli & Heausgeber. Cham: Springer International Publishing, 2020 [zitiert 24. December 2024]. Verfügbar unter. (10.1007/978-3-030-24243-5_17)

[47] Abhari S, Hsing TM, Malkani MM, et al. Patient satisfaction following total knee arthroplasty using restricted kinematic alignment. Bone Joint Lett J 2021 103-B (6 Supplement A) 59–66. (10.1302/0301-620x.103b6.bjj-2020-2357.r1) 34053299

[48] Vendittoli PA, Martinov S & Blakeney WG. Restricted kinematic alignment, the fundamentals, and clinical applications. Front Surg 2021 8 697020. (10.3389/fsurg.2021.697020) 34355018 PMC8329359

[49] MacDessi SJ. Restricted kinematic alignment in total knee arthroplasty: scientific exploration involving detailed planning, precise execution, and knowledge of when to abort. Arthroplast Today 2021 10 24–26. (10.1016/j.artd.2021.05.024) 34277907 PMC8267482

[50] MacDessi SJ, Griffiths-Jones W, Chen DB, et al. Restoring the constitutional alignment with a restrictive kinematic protocol improves quantitative soft-tissue balance in total knee arthroplasty: a randomized controlled trial. Bone Joint Lett J 2020 102-B 117–124. (10.1302/0301-620x.102b1.bjj-2019-0674.r2) PMC6974544 31888372

[51] MacDessi SJ, Griffiths-Jones W, Harris IA, et al. The arithmetic HKA (aHKA) predicts the constitutional alignment of the arthritic knee compared to the normal contralateral knee: a matched-pairs radiographic study. Bone Jt Open 2020 1 339–345. (10.1302/2633-1462.17.bjo-2020-0037.r1) 33215122 PMC7659698

[52] Chang JS, Kayani B, Wallace C, et al. Functional alignment achieves soft-tissue balance in total knee arthroplasty as measured with quantitative sensor-guided technology. Bone Joint Lett J 2021 103-B 507–514. (10.1302/0301-620x.103b.bjj-2020-0940.r1) 33467917

[53] Choi BS, Kim SE, Yang M, et al. Functional alignment with robotic-arm assisted total knee arthroplasty demonstrated better patient-reported outcomes than mechanical alignment with manual total knee arthroplasty. Knee Surg Sports Traumatol Arthrosc 2023 31 1072–1080. (10.1007/s00167-022-07227-5) 36378291

[54] Clark G, Steer R & Wood D. Functional alignment achieves a more balanced total knee arthroplasty than either mechanical alignment or kinematic alignment prior to soft tissue releases. Knee Surg Sports Traumatol Arthrosc 2023 31 1420–1426. (10.1007/s00167-022-07156-3) 36116071 PMC10050049

[55] Kafelov M, Batailler C, Shatrov J, et al. Functional positioning principles for image-based robotic-assisted TKA achieved a higher forgotten joint score at 1 year compared to conventional TKA with restricted kinematic alignment. Knee Surg Sports Traumatol Arthrosc 2023 31 5591–5602. (10.1007/s00167-023-07609-3) 37851026

